# Minimum Latency-Secure Key Transmission for Cloud-Based Internet of Vehicles Using Reinforcement Learning

**DOI:** 10.1155/2022/6296841

**Published:** 2022-09-26

**Authors:** V. Akilandeswari, Ankit Kumar, S. Thilagamani, V. Subedha, V. Kalpana, Kiranjeet Kaur, Evans Asenso

**Affiliations:** ^1^Department of Information Technology, Sethu Institute of Technology, Virudhunagar, Tamil Nadu, India; ^2^Department of Computer Engineering and Applications, GLA University, Mathura, Uttar Pradesh, India; ^3^Department of Computer Science and Engineering, M. Kumarasamy College of Engineering, Thalavapalayam, Karur, Tamilnadu, India; ^4^Department of CSE, Panimalar Institute of Technology, Pidarithangal, Tamil Nadu, India; ^5^Department of Computer Science and Engineering, K. Ramakrishnan College of Technology, Samayapuram, Trichy, Tamilnadu, India; ^6^Department of CSE, University Centre for Research & Development, Chandigarh University, Mohali, Punjab 140413, India; ^7^Department of Agricultural Engineering, School of Engineering Sciences, University of Ghana, Accra, Ghana

## Abstract

The Internet of Vehicles (IoV) communication key management level controls the confidentiality and security of its data, which may withstand user identity-based attacks such as electronic spoofing. The IoV group's key is updated with a defined frequency under the current key management method, which lengthens the time between crucial changes and encryption. The cluster key distribution management is used as the study object in this paper, which is based on the communication security on the Internet of Vehicles cluster. When vehicles enter and exit the cluster, the Internet of Vehicles must update the group key in real-time to ensure its forward and backward security. A low-latency IoV group key distribution management technology based on reinforcement learning is proposed to optimize the group owner vehicle according to factors such as changes in the number of surrounding vehicles and essential update records and the update frequency and the key length of its group key. The technology does not require the group leader vehicle to predict the nearby traffic flow model. The access-driven cache attack model reduces the delay of encryption and decryption and is verified in the simulation of the IoV based on advanced encryption standards. The simulation results show that, compared with the benchmark group key management scheme, this technology reduces the transmission delay of key updates, the calculation delay of encryption and decryption of the IoV, and improves the group key confidentiality.

## 1. Introduction

The shared group key in the vehicle network (VANET, vehicular ad hoc networks) cluster performs symmetric encryption on the communication of group members [1], which is the key to ensuring the communication security and user privacy in the process of cooperative driving of cluster vehicles, congestion avoidance, and entertainment services [2, 3]. Due to the high node mobility, VANET is a specific type of wireless multicast network that must accommodate quick topology changes [4]. Intervehicle connectivity is developing into a potential area for investigation, standardisation, and growth as more cars are outfitted with wireless communication and computing technology. A subclass of mobile ad hoc networks (MANETs) and vehicular ad hoc networks (VANET) are networks that are created by moving vehicles [5]. It can be put into systems that are connected to health, where it can save a lot of lives every day, and nonsecurity applications for commercial purposes. However, due to the dynamic and open nature of the Internet of Vehicles (IoV) cluster, the cluster is vulnerable to various internal and external network attacks. IoV systems are subject to a variety of assaults, including those on authenticating and identity, availability, secrecy, routing, and data validity, which leads to a number of difficult security and privacy requirements. In recent years, various security researchers have worked hard to secure the privacy and security of the Internet of Vehicles. Typical assaults on accessibility include denial-of-service and channel interference. This kind of attack primarily makes use of the bandwidth and transmission power restrictions to bring down the IoV system [6, 7].

Attackers use timing attacks, cache attacks [8], and other means to steal the group key, conduct eavesdropping, electronic spoofing, and other attacks, resulting in user privacy: spills and traffic accidents. Therefore, the Internet of Vehicles needs to update the group key in real-time to ensure the forward and backward security of the group key when vehicles join and leave the cluster [9].

While the IoV cluster uses the group key to perform node authentication and communication encryption for vehicles, which has attracted extensive attention, how to effectively manage the IoV key is an urgent problem that needs to be solved. Reference [10] proposes an anonymous key management scheme for the Internet of Vehicles based on critical public infrastructure (PKI, public key infrastructure), which authenticates vehicle nodes and protects data integrity through anonymous digital certificates. However, keys and anonymous certificates make the certification centre pay a high management cost to ensure system security and impose a burden on vehicle node storage and communication overhead. Reference [11] proposes using cluster-based peer-to-peer batch key agreement, but vehicle nodes can only belong to one cluster. When a new node joins the cluster, the cluster leader needs to renegotiate keys with cluster members, which is challenging to apply to high dynamic Sexual Internet of Vehicles. Researchers have developed a STRIDE model for data security that groups security threats into sexual categories including spoof, tampering, repudiation, and information disclosure. The IoV system is susceptible to the threats outlined above and can be targeted in a variety of ways, including network jamming, eavesdropping, and infiltration. The IoV system could be harmed by these attacks, which could weaken its resistance and reliability or, in the worst case, bring it to a standstill and result in accidents. The GKA scheme proposed by Reference [12] uses pseudonyms for cluster authentication and key update. It uses aliases to construct digital signatures while simultaneously authenticating cluster vehicles to prevent the leakage of vehicle trajectory privacy. Reference [9] proposes to use a software-defined network to manage the Internet of Vehicles. Different clusters independently build a decentralized network for communication and use a significant network to authenticate and collect the keys of the groups. Reference [13] proposed to perform distributed batch anonymous authentication on vehicles by computing the hash message authentication code instead of the authentication revocation list for the clustered decentralized network. This scheme protects user privacy and reduces the authentication delay; Feriani and Hossain [14]. It is proposed that based on the trust mechanism, after the vehicle with a high reputation is certified by the trust centre, anonymous key negotiation can be carried out to ensure the confidentiality and integrity of the message while protecting privacy.

As the leading research direction of machine learning, reinforcement learning is widely used in vehicle networking communication security [15, 16]. Security is a key challenge because the Internet of Things has such a significant impact on its consumers' lives. By enabling hackers to gain direct control of automobiles, a failure in the IoV system could endanger safety and could result in road accidents. In an IoT setup, like a smart grid, all sensors, gadgets, and machines can effectively and safely control energy use while humans act as observers. While inside the car, automotive monitors like brake sensors, limited fuel, and tyres pressure sensors, among others, are fitted, and the exterior of the car has security mechanisms like CCTV and parking sensors. Reference [17, 18] proposed using reinforcement learning to assist mobile devices in optimizing relay scheme, transmit power, and communication frequency to resist hostile interference. This scheme can reduce energy consumption and ensure communication reliability. In the case of the unknown channel model and spoofing attack model, Reference [19, 20] uses reinforcement learning to optimize the authentication threshold of wireless channel physical layer characteristics such as received signal strength and achieve high-precision authentication in the dynamic game of malicious senders. Reference [21] used reinforcement learning-based on the long-term memory effect for joint user access control and battery energy prediction, improving network throughput while saving energy. Reference [22] proposes using reinforcement learning to perform joint buffer allocation and interference alignment according to the current buffer usage and channel state to solve multi-user interference in wireless communication and improve the transmission rate.

This paper is based on the communication security on the Internet of Vehicles cluster and takes the cluster key distribution management as the research object. In this network, vehicles with common interests in the same area (such as destinations, hobbies, and friend groups) build clusters, cooperate to form driving patterns based on vehicle queues, share road conditions and driving information, improve with a small and constant distance between vehicles road capacity and energy efficiency, and provide multimedia services. Since vehicles frequently join and leave the cluster, to ensure the safety of cars in the group and prevent electronic spoofing and other attacks, some information in the cluster, such as road conditions and multimedia needs to be updated in real-time. Compared with traditional PKI, this paper proposes a certificateless signature scheme, which abandons digital certificates and signs the group key through a random prime number secretly stored between the trust centre and the vehicle to ensure data integrity. The unit (RSU, roadside unit) broadcasts the group key update information, realizes the synchronous update of the group key, reduces the communication overhead of the vehicle node updating the group key, and reduces the transmission delay of the group key update. Currently, services for the Internet of Vehicles are mostly provided through cloud computing technology. A computer model known as “cloud computing” is one in which customers have 24/7 access to the Internet and computing capabilities are offered on a pay-as-you-go basis. Because of the IoV's limitations in terms of bandwidth, transmission power, and mobility, IoV routing algorithms are typically somewhat complicated. This complexity causes holes and weaknesses in the IoV routing method as a result. When the group leader's update strategy has no previous effect, the update strategy of the group key is only related to the current state of the cluster, so the optimization process of the critical update strategy is constructed as a Markov decision process. This paper proposes a low-latency group key distribution management technology based on reinforcement learning to obtain the optimal required update frequency and critical length in a dynamic cluster environment. Under the premise, the technology uses reinforcement learning to optimize the update frequency and crucial length of the group key according to the number of cluster vehicles, the change in the number of cluster vehicles, the binding update decision in the previous period, and the security level of the current cluster communication. In the learning of swarm communication, the optimal key update strategy is obtained, the computational delay of encryption and decryption of cluster communication and the probability of the group key being stolen are reduced, and the quality of cluster communication service is improved.

## 2. System Model

### 2.1. Key Management Model

Key production, distribution, usage storing, rotation, backup or recovery, and revocation destruction are all steps in the lifecycle of activities that make up key management model. When used in combination with 5G and beyond, AI technologies like machine learning (ML) and reinforcement learning (RL) offer special alternatives to dependability issues in low-latency vehicle communication networks. Numerous IoV applications demand adaptable and clever resources systems. Applications for information services, obstacle detection, and accident prevention, for instance, the need for information exchange through regular access to services with a high data transmission rate [23, 24]. The low-latency key management technology of the Internet of Vehicles is mainly composed of an On-Board unit (OBU), a roadside unit, and a Trusted Authority (TA) assembled in the vehicle. The OBU communicates with the RSU and surrounding vehicles using the dedicated short-range communication (DSRC) protocol [25]. RSU is really impenetrable and secure. RSU is unable to sign delivered messages simultaneously in the names of OBU and LCA. It is assumed in this analysis that RSUs have no computational, energy, or capacity constraints. RSUs can therefore serve as middlemen for the transmission of communications from OBUs. Because OBUs and RSUs can communicate wirelessly, each vehicle must have a tamper-proof device installed. Security, encryption, and decryption operations can be carried out by OBU. Moving vehicles' On-Board units (OBUs) communicate information such as location, the current situation, traffic, speed, direction, and accident occurrences, continually and occasionally. The aforementioned data will be processed, gathered, and provided to the cloud platform and drivers using RSUs. As a communication relay and broadcast node, RSU connects with TA through a wired network and communicates with OBU through a wireless network. When all vehicles enter the TA registration for the first time, the TA distributes the vehicle identification ID, generates a random prime number *n* for developing the group key and encrypted group key, generates the public key VPK and the private key VSK, and registers {ID, VPK, *n*}, and store {ID, VSK, *n*} in the OBU of the vehicle. In addition, TA registers RSU's identity RID and its public key RPK and stores its private key RSK in RSU.  Table 1 shows the important parameter symbols.

**Figure 1:**
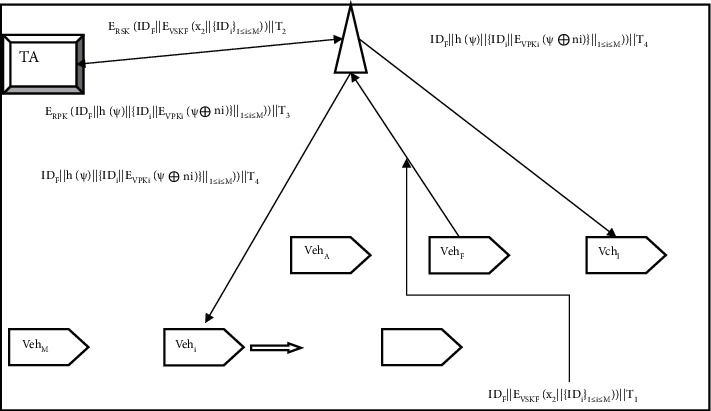
Key management model.

As shown in Figure 1, the Internet of Vehicles organizes *M* vehicle nodes {Veh_*i*_}_1≤*i*≤*M*_ to build a cluster in a particular area based on a dynamic clustering algorithm [26] and provides them with services such as cooperative driving and congestion avoidance. The moving vehicles joining and leaving the cluster have certain randomness, so the group key needs to be updated in real-time. It is assumed that the number of vehicle nodes entering and leaving the set in unit time obeys a Poisson distribution with mean *λ*, and the cluster vehicle identity is {ID_*i*_}_1≤*i*≤*M*_. To ensure the security of communication and privacy within the cluster, a symmetric encryption mechanism is used for intracluster communication. In addition, a certificateless signature key management scheme is used to update the group key and cluster head (cluster head) Veh_*F*_ for the cluster. The tremendous growth in the smart automotive sectors has recently led to a huge rise in demand in Internet of Vehicles (IoV) technology. Vehicles can interact with their surroundings and public networks thanks to Internet of Vehicles technologies. Additionally, it enables moving objects to communicate and gather data on other moving objects and highways. The group key needs to be updated in real-time since the moving vehicles joining and departing the cluster have some degree of randomness. The cluster's group key and cluster head (cluster head) are updated using a key management mechanism. The IoV group's key is updated with a fixed frequency under the current key management method, which lengthens the time between critical updates and encryption.

The RSU is responsible for the secondary encryption of the request and forwards it to the TA, and the TA feeds back the new key according to the request. Specifically, the cluster leader Veh_*F*_ observes the number of vehicles in the cluster, the number of vehicles joining and leaving the group, the average driving speed of the collection, the critical update decision at the last *D* moments and the security level at the current moment, and generates a key update request. The binding update request mainly contains the cluster vehicle ID of the updated key and the length of the new key *x*_2_, namely, *x*_2_‖{ID_*i*_}_1≤*i*≤*M*_. Veh_F_ encrypts the critical update request with the private key VSK_F_, adds a timestamp *T*_1_ and sends it to the RSU. After the RSU receives the necessary update request at *T*_1_^*∗*^, it calculates *T*_1_^*∗*^ − *T*_1_, which is used to verify the validity of the binding update request and prevent replay attacks. If *T*_1_^*∗*^ − *T*_1_ < Δ*T*_1_, the RSU uses the private key RSK to encrypt the key update request again and sends the key update request to the TA through the wired network.

TA receives the key update request forwarded by the RSU and first calculates if *T*_2_^*∗*^ − *T*_2_ < Δ*T*_2_ to verify the validity of the key update request. The TA decrypts the key update request using the public keys RPK and VPK_F_ and obtains the new key length *x*_2_ and identity {ID_*i*_}_1≤*i*≤*M*_, while authenticating Veh_F_ and RSU. According to {ID_*i*_}_1≤*i*≤*M*_ corresponding to {*n*_*i*_}_1≤*i*≤*M*_, TA uses the HCPA-GKA [27] algorithm based on the Chinese remainder theorem to generate a group key *ψ* of length *x*_2_ and calculate the hash The value *h*(*ψ*) generates a digital signature. TA uses {*n*_*i*_}_1≤*i*≤*M*_ and *ψ* to perform XOR operation to encrypt the group key, and uses {VPK_*i*_}_1≤*i*≤*M*_ to encrypt the XOR result to ensure the security of key transmission, that is, to generate *h*(*ψ*)‖{*E*_VPK_*i*__(*ψ* ⊕ n_i_)}_1≤*i*≤*M*_. TA encrypts the ID_*F*_‖*h*(*ψ*)‖{*E*_VPK_*i*__(*ψ* ⊕ n_i_)}_1≤*i*≤*M*_ with RPK and sends it to the RSU, which decrypts it and broadcasts it to the swarm vehicle {Veh_*i*_}_1≤*i*≤*M*_.

The group member Veh_*i*_ receives the critical update message by RSU and uses the private key VSK_*i*_ to decrypt the *E*_VPK_*i*__(*ψ* ⊕ *n*_*i*_) part corresponding to ID_i_ to obtain *ψ* ⊕ *n*_*i*_. Then, the group members use the random prime number *n*_*i*_ stored in the OBU to decrypt *ψ*^*∗*^=*ψ* ⊕ *n*_*i*_ ⊕ *n*_*i*_ to obtain the group key *ψ*^*∗*^ and calculate its hash value *h*(*ψ*^*∗*^) to verify the authenticity of the new key *ψ*^*∗*^.

If *h*(*ψ*^*∗*^) ≠ *h*(*ψ*), the received vital update information is a forged message; otherwise, update *ψ*^*∗*^ as a new key for intracluster communication.

### 2.2. Attack Model

Attack graph creation computation time begins when network complexity increases proportionally in a linear path to the number of subnets. The length of time it takes to generate an attack graph varies with network size. This paper mainly considers the internal attack on the Internet of Vehicles. The attacker can be a legitimate vehicle node in the network, or a controlled malicious vehicle node, which obtains the group key through side-channel attacks such as timing attacks and cache attacks [28], and then implements an electronic spoofing attack [29]. The act of spoofing involves hiding a message or identification so that it looks to be coming from a reliable, approved source. Sensitive personal or business data may be stolen, credentials may be gathered for use in fraud or future attacks, malware may be transmitted via harmful links or attachments, trust relationships may be used to gain unwanted network access, and access limits may be disregarded. They may even conduct a man-in-the-middle (MITM) assault or a denial-of-service (DoS) attack. The effect of IoV interactions among RSUs, cars, and other TPMs is implied by the routing algorithm and its quality, and IoV routing methods are usually rather complex due to the IoV's limits of bandwidth, transmission power, and mobility. As a result, this complication creates gaps and vulnerabilities in the IoV routing mechanism. When an attacker pretends to be a legitimate equipment or user to steal information, transmit malware, or get around security systems, this is called spoofing. Man-in-the-middle (MITM), in this instance, the attacker places himself in the middle of two clients that are speaking and spoofs both of their addresses. By doing this, each victim transmits a data packet to the hacker rather than directly to its intended recipient. A Denial-of-Service (DoS) attack aims to bring down a computer system or network so that its targeted recipient is unable to access it. DoS attacks achieve this by providing the victim with an excessive amount of traffic or content that causes a crash. More specifically, attackers use spyware to observe the delay side-channel information during the encryption process, monitor cache hits and misses, and steal encryption keys. The attacker pretends to be an authenticated vehicle or RSU and sends malicious or forged information, such as traffic density and collision avoidance information, to surrounding vehicles through legal identities. Even cars pretending to be legal entities send this information to surrounding vehicles.

Assuming that the probability of a successful internal attack by each vehicle node is *y*, the chance of being attacked by a cluster with *M* vehicles is 1 − (1 − *y*)^*M*^. Among them, the probability of a successful cluster attack is positively correlated with the number of cluster vehicles and critical update frequency and negatively correlated with the key length.

## 3. Key Management Technology Based on Reinforcement Learning

Different models, classifications, and training approaches for machine learning are frequently utilized for prediction issues and intelligent management. Reinforcement learning (RL) will give behavior guidance in IoV applications to increase resilience and scalability. In IoV networks, it can provide routes from source to destination or route optimization. Wireless local area networks (WLANs) will gain new capabilities to meet user needs from flexible network structures, such as the software-defined networking (SDN) model while attaining higher levels of effectiveness and mobility in those complicated circumstances. By identifying workable configurations through learning, machine learning (ML) approaches used in conjunction with SDN may enhance network resource consumption and management. Throughput maximisation and latency reduction may be guaranteed with the use of machine learning (ML) and the Software-Defined Network (SDN) in the Internet of Things (IoV). By offering more dependable and enhanced routing services, ML and SDN will improve IoV network performance. A distribution cache replacement approach based on the popularity of the material will be possible with a reinforcement learning scheme like the Q-learning technique. It also has the ability to predict the unknowable popularity of cached contents. With the joining and leaving of vehicles, the merging and splitting of the cluster, and the change of communication security level, the group leader needs to update the group key of the intracluster communication in real-time [30, 31]. The dynamic optimization process of the required length and update frequency of the group key, the security level of the group communication, and the calculation delay of communication encryption and decryption can be regarded as a Markov process. This paper proposes a low-latency IoV group key distribution management technology based on reinforcement learning. The group leader optimizes the length and update frequency of the group key and obtains the optimal key update strategy through continuous trial and error and knowledge.

In this technique, the cluster performs intrusion detection based on physical layer characteristics, such as channel status and received signal strength [32, 33], and Veh_*F*_ evaluates the communication security level of the cluster-based on the feedback detection results *ρ*. When the cluster detects that the vehicle is under attack, such as spoofing, *ρ* = 0; otherwise, *ρ* = 1. The cluster leader Veh_*F*_ comprehensively considers information such as the number of vehicles in the cluster *M*, the cluster average speed *v*, the critical update decision in the first *D* moments and the security level *ρ* of the cluster communication at the last moment, and selects the critical update according to the long-term learning benefit of the cluster [34, 35]. The policy *x* ∈ *A* includes whether to send a key update request *x*_1_ ∈ {0, 1} and the updated vital length *x*_2_ ∈ [1, *L*_*A*_] bit, that is, *x* = [*x*_1_, *x*_2_]. Among them, *L*_*A*_ is the upper limit value of the critical length, and *A* is the essential management action space. When *x*_1_ = 0, Veh_*F*_ does not send a key update request, and the cluster continues to use the previous key for communication encryption; when *x*_1_ = 1, Veh_*F*_ requests the TA to update the group key of length *x*_2_.

At time *k*, Veh_*F*_ observes the number of vehicles *M*^*k*−1^ and *M*^*k*^ at time *k* − 1 and time *k* in the cluster, the average speed of the cluster vehicles , the critical update decision *g*^*k*^=[*x*_1_^*i*^]_*k*−*D*≤*i*≤*k*−1_ and the security level *ρ*^*k*−1^ of the cluster communication at time *k* − 1, and construct the current cluster state *s*^*k*^.

(1) $$\begin{array}{c} s^{k}=\left[M^{k-1},M^{k},v^{k},g^{k},\rho^{k-1}\right]. \end{array}$$

Let *Q*(*s x*) denote the long-term payoff of Veh_*F*_ taking action *x* in state *s*; Veh_F_ selects a more efficient group key by using an *ε*-greedy strategy based on the current cluster state *s*^*k*^ through the corresponding *Q* value. New strategy *x*^*k*^=[*x*_1_^*k*^, *x*_2_^*k*^]. Specifically, Veh_*F*_ selects the key update strategy with the largest long-term benefit *Q*(*s*^*k*^, *x*) in the current state *s*^*k*^ with the probability of 1 − *ε*; arbitrarily selects a key update strategy from *A* with the probability of *ε*, namely,

(2) $$\begin{array}{c} p\left(x^{k}=x^{*}\right)=\left\{\begin{array}{cc} 1-\epsilon \text{,} & x^{*}=\underset{x\in A}{argmax}Q\left(S^{k},x\right)\text{,}\\ \frac{\epsilon }{\left|A\right|}\text{,} & \forall x^{*}\in A\text{.} \end{array}\right. \end{array}$$

At time *k*, if Veh_*F*_ sends a key update request forwarded by RSU to TA, the request contains cluster vehicle identity {*ID*_*i*_}_1≤*i*≤*M*_ and key length *x*_2_^*k*^. TA receives the critical update request and generates a group key *ψ* is returned to the RSU; after the RSU receives the encrypted new key, it broadcasts it to the cluster vehicles to update the entire cluster key.

When the cluster performs a critical update, Veh_*F*_ obtains the current security level *ρ*^*k*^ of intra

cluster communication through the feedback of cluster members and evaluates the calculation of cluster encryption and decryption of the group key of length *x*_2_^*k*^. Veh_F_ evaluates the benefit *u*^*k*^ of this critical update by the number of cluster vehicles *M*^*k*^, the cluster communication security level *ρ*^*k*^, and the essential length *x*_2_^*k*^.

(3) $$\begin{array}{c} u^{k}=\rho^{k}M^{k}-c_{1}x_{2}^{k}M^{k}-c_{2}x_{1}^{k}M^{k}. \end{array}$$

Among them, *c*_1_ and *c*_2_ represent the weights of the computation delay and critical update delay of cluster communication encryption and decryption, respectively.

Veh_*F*_ observes the state *s*^*k*+1^ at the time of cluster *k* + 1 and updates the *Q* value corresponding to the state-action pair (*s*^*k*^, *x*^*k*^) based on the Bellman equation.

(4) $$\begin{array}{c} Q\left(s^{k},x^{k}\right)\leftarrow \left(1-\alpha \right)Q\left(s^{k},x^{k}\right)+\beta \left(u^{k}+\max_{x\in A}Q\left(s^{k+1},x^{k}\right)\right). \end{array}$$

The specific process of the IoV key management technology based on reinforcement learning is shown in Algorithm 1.

## 4. Simulation Experiments

3D technology is now a prominent technology that is desired and used in a variety of sectors. The current standard traffic simulation software has a scene interface with an excellent 3D effect, which allows users to generate simulation scenarios more logically and stereoscopically. Trans-Modeler, a simulation programme that enables 3D modelling, offers an amazing 3D impact when 3D models must be addressed during modelling. This paper uses Python to build a dynamic cluster simulation scenario to verify the advantages of the low-latency IoV group key distribution management technology based on reinforcement learning in the computing delay and communication security level of encryption and decryption. Reference [29] for the setting of stimulation parameters are as follows: the group leader and vehicles within a range of 300 m form a cluster, the number of vehicle nodes in the initial cluster is 50, and the average speed of vehicles in the cluster *v* ∈ [0,120] km/h, and the same as that of the cluster vehicles. The number is inversely proportional [30]. Cluster vehicles use the Advanced Encryption Standard (AES) symmetric encryption algorithm to encrypt intracluster communication, with optional key length *x*_2_ ∈ {128,192,256} bits. The packet size for intracluster vehicle communication is 1 kB. The learning rate of *Q*-learning is *α* = 0.2, and the discount factor *β* = 0.8.

**Figure 2:**
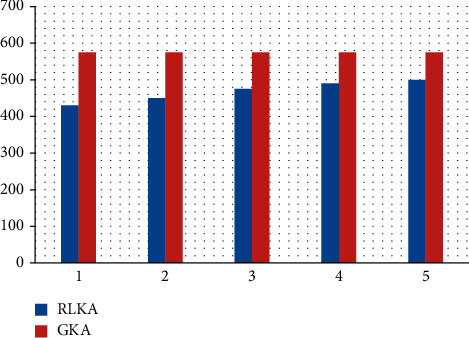
Calculation delay of vehicle encryption and decryption in the cluster w.r.t. time slot.

**Figure 3:**
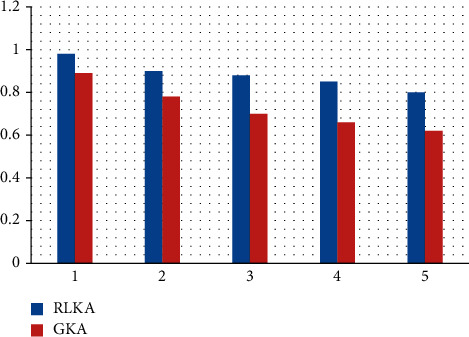
Cluster communication security level w.r.t. time slot.

Figure 2 and Table 2 provide the average performance of reinforcement learning-based key management (RLKA) over 3000-time slots, examining the average number of vehicles joining and leaving the cluster per time slot *λ* ∈ {1, 2,…, 5}.  Figure 3 and Table 3 depict the effect of encryption and decryption calculation delay and cluster communication security level. It is clear from Figures 2 and 3 that when *λ* increases from 1 to 5, the calculation delay of vehicle encryption and decryption of 1 kB data packet in the cluster increases from 432 *μ*s increased to 497 *μ*s, the latency increased by 15.0%, and the cluster communication security level decreased from 0.963 to 0.794, and the performance decreased by 17.5%. When *λ* = 3, compared with the GKA scheme, the proposed technique's encryption and decryption calculation delay are reduced by 18.1%, and the security level is improved by 24.1%. This is because, compared with the fixed vital length and update frequency of the GKA scheme, the RLKA technology proposed in this paper can be based on the number of vehicles in the current cluster, the number of vehicles joining and leaving the group, and the safety feedback of cluster vehicle intrusion detection [31]. Prescan simulation software uses a basic vehicle dynamics model, which is unable to drive the intelligent vehicle precisely in the vertical direction or to effectively reflect the vehicle's dynamic properties in that direction. CarSim simulation tool is advised when the necessary study incorporates vehicle dynamics. In many ways, the domestic traffic flow operation law differs from that of other nations, particularly when the signal light is yellow because of differences in driver behavior and vehicle operation. When a driver sees a yellow light in the real world, he is more likely to speed through the junction than to stop and slow down. The software programmes, like Vissim and Prescan, do take this into account but do not set a variable driving reaction model, and the simulation result is quite different from the real scenario [32]. Therefore, reasonably update of the key and control of the length of the key, reduce the probability of successful attack by malicious nodes, ensure the security of IoV cluster communication, and obtain minor encryption and decryption calculation delay overhead. Since the IoV cluster requires the cluster key to conduct node identification and connection cryptography for automobiles, which has garnered considerable attention, it is urgently necessary to find a solution to the issue of how to maintain the IoV key. The routing algorithm and its quality indicate the impact of IoV interactions between RSUs, automobiles, and other TPMs, and IoV routing techniques are typically somewhat complicated because to the IoV's bandwidth limitations. The foundational framework for creating a smart IoV environment is provided by the 5G network. In order to reach high performance, it pushes the vehicle network capabilities. The Internet of Everything (IoE) is capable of a new evolution thanks to 5G technology [36].

**Figure 4:**
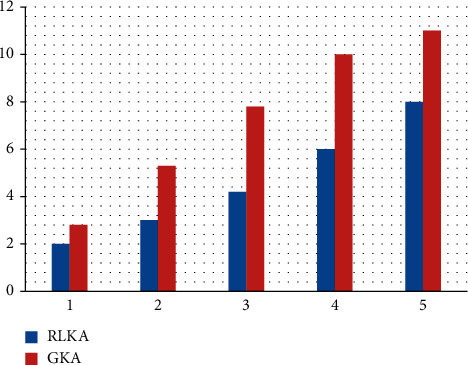
Key update transmission delay.

To verify the advantage of RLKA technology using certificates signature in terms of communication overhead, this paper uses MATLAB to build a simulation of the transmission delay of essential updates. Reference [13] for simulation parameter setting, the wireless transmission rate between vehicle node and RSU is 600 Mbit/s, and the data size of vehicle ID, timestamp *T*, and request key length *x*_2_ is 4 bytes. As shown in Figure 4 and Table 4, when the number of cluster vehicles increases from 20 to 100, due to the increase in the data volume of critical update information, the transmission delay of cluster key update information rises linearly with the number of cluster vehicles. When the number of cluster vehicles is 60, compared with the GKA [16] scheme, the critical update transmission delay of the proposed technique is reduced by 31.0%. Compared with the PKI-based key management scheme of the Internet of Vehicles, this paper uses the certificateless signature key management technology, abandons the digital certificate, avoids the communication delay and communication load caused by the transmission of the digital certificate, and realizes the group key through RSU broadcast.

## 5. Conclusion

This paper proposes a low-latency IoV group key distribution management technology based on reinforcement learning. The group leader vehicle observes cluster characteristics such as the number of surrounding vehicles and vehicle speed. It combines the critical update decision at the last moment with the current IoV communication security. Construct the cluster state according to the situation, use reinforcement learning to optimize the update frequency and critical length of the group key, realize the resistance to attacks such as crucial theft and spoofing through low-latency cluster key update, improve the security level of cluster communication, and reduce the number of intracluster attacks—communication delay. Researchers were having trouble figuring out how to apply deep learning methodologies to IoV applications due to issues with big data simulations, control computation, and resource management. These issues call for significant improvements in order to create a coherent system that satisfies both service excellence and customer quality requirements. From this research, the simulation results demonstrate that, when compared to the GKA scheme, the proposed RLKA technology can guarantee communication security while also lowering the transmission delay of group key updates, lowering the computational uncertainty of communication encryption and decryption within the cluster, enhancing the confidentiality of the group key, and enhancing the quality of cluster communication services.

## Figures and Tables

**Algorithm 1:**
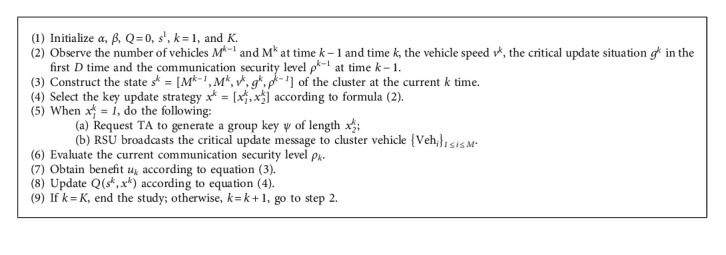
Low-latency IoV key distribution management algorithm based on reinforcement learning.

**Table 1:** Important parameter symbols.

Parameter	Meaning
*M* ^*k*^	The number of vehicles in the cluster at time *k*
*v* ^*k*^	The average speed of cluster vehicles
*g* ^*k*^	Cluster critical update decisions for the first *D* moments
*ρ* ^*k*^	Cluster communication security level
*x*_1_^*k*^	Update key decision
*x*_2_^*k*^	Update key length
*λ*	Poisson distribution mean
VPK/VSK	Vehicle's public/private key
RPK/RSK	RSU public/private key
*n*	Random prime is chosen by TA
*E*(·)	Encryption
*h*(·)	Hash function
*T*/*T*^*∗*^	Send/receive timestamp

**Table 2:** Calculation delay of vehicle encryption and decryption.

Algorithm	Time slot 1	Time slot 2	Time slot 3	Time slot 4	Time slot 5
RLKA	430	450	475	490	500
GKA	575	575	575	575	575

**Table 3:** Cluster communication security level.

Algorithm	Time slot 1	Time slot 2	Time slot 3	Time slot 4	Time slot 5
RLKA	0.98	0.9	0.88	0.85	0.8
GKA	0.89	0.78	0.7	0.66	0.62

**Table 4:** Transmission delay with increasing the number of vehicles in a cluster denoted by *N*.

Algorithm	*N* = 20	*N* = 40	*N* = 60	*N* = 80	*N* = 100
RLKA	2	3	4.2	6	8
GKA	2.8	5.3	7.8	10	11

## Data Availability

The data shall be made available on request.
